# Preconditioning Involves Selective Mitophagy Mediated by Parkin and p62/SQSTM1

**DOI:** 10.1371/journal.pone.0020975

**Published:** 2011-06-08

**Authors:** Chengqun Huang, Allen M. Andres, Eric P. Ratliff, Genaro Hernandez, Pamela Lee, Roberta A. Gottlieb

**Affiliations:** The BioScience Center, San Diego State University, San Diego, California, United States of America; Instituto de Química - Universidade de São Paulo, Brazil

## Abstract

Autophagy-dependent mitochondrial turnover in response to cellular stress is necessary for maintaining cellular homeostasis. However, the mechanisms that govern the selective targeting of damaged mitochondria are poorly understood. Parkin, an E3 ubiquitin ligase, has been shown to be essential for the selective clearance of damaged mitochondria. Parkin is expressed in the heart, yet its function has not been investigated in the context of cardioprotection. We previously reported that autophagy is required for cardioprotection by ischemic preconditioning (IPC). In the present study, we used simulated ischemia (sI) in vitro and IPC of hearts to investigate the role of Parkin in mediating cardioprotection ex vivo and in vivo. In HL-1 cells, sI induced Parkin translocation to mitochondria and mitochondrial elimination. IPC induced Parkin translocation to mitochondria in Langendorff-perfused rat hearts and in vivo in mice subjected to regional IPC. Mitochondrial depolarization with an uncoupling agent similarly induced Parkin translocation to mitochondria in cells and Langendorff-perfused rat hearts. Mitochondrial loss was blunted in Atg5-deficient cells, revealing the requirement for autophagy in mitochondrial elimination. Consistent with previous reports indicating a role for p62/SQSTM1 in mitophagy, we found that depletion of p62 attenuated mitophagy and exacerbated cell death in HL-1 cardiomyocytes subjected to sI. While wild type mice showed p62 translocation to mitochondria and an increase in ubiquitination, Parkin knockout mice exhibited attenuated IPC-induced p62 translocation to the mitochondria. Importantly, ablation of Parkin in mice abolished the cardioprotective effects of IPC. These results reveal for the first time the crucial role of Parkin and mitophagy in cardioprotection.

## Introduction

Cellular homeostasis depends upon adequate protein and organelle quality control. Cells have evolved selective clearance mechanisms to eliminate misfolded and aggregated proteins or dysfunctional organelles. Molecular chaperones in the heat shock family of proteins facilitate the refolding of misfolded proteins. Unrepaired misfolded proteins are ultimately targeted for degradation by the ubiquitin-proteasome system (UPS) [1], [2]. Elimination of larger dysfunctional complexes, aggregated proteins or organelles such as mitochondria is accomplished via autophagy [2], [3], [4], [5].

Currently, there is great interest in determining how autophagy can selectively recognize and remove damaged mitochondria. Several groups have shown that in neuronal cells, Parkin is involved in the selective targeting and degradation of damaged mitochondria through autophagy (mitophagy) [5], [6], [7], [8], [9]. Loss of mitochondrial membrane potential (ΔΨ_m_) stabilizes mitochondrial outer membrane-associated protein PINK1, a kinase that signals Parkin recruitment to the mitochondria [5], [6], [7]. Parkin is an E3 ubiquitin ligase found in the liver, kidneys, testis, brain, skeletal muscle, and heart [10], [11]. Under basal conditions, Parkin is mainly found in the cytosol where its ubiquitin ligase activity is inhibited by an unknown mechanism [6]. However, under physiologic and pathologic stress, Parkin translocates to damaged mitochondria in a PINK1-dependent manner to ubiquitinate mitochondrial proteins such as VDAC1, Mfn1, Mfn2 and other proteins [3], [12], [13], [14].

Recently, p62/SQSTM1/sequestosome-1 (referred to as p62 hereafter) has been shown to bind ubiquitinated mitochondria through its ubiquitin binding domain, and to recruit the phagophore (cup-shaped early stage autophagosome) through an LC3 binding domain [15], [16], [17], [18]. Ablation of p62 has been shown to attenuate the degradation of mitochondria compromised by chemical uncouplers that dissipate the mitochondrial membrane potential, revealing its role in mitophagy [3], [19], although this is controversial [20], [21]. We previously reported that ischemic preconditioning in isolated rat hearts mediates cardioprotection against I/R injury through an autophagy-dependent pathway involving p62 recruitment to mitochondria [22]. IPC has been shown to involve opening of the mitochondrial permeability transition pore [23], and we have demonstrated a role for the permeability transition pore in mediating mitophagy in starved HL-1 cells [24]. For these reasons, we sought to determine if Parkin plays a role in IPC-induced cardioprotection. Here we demonstrate that Parkin mediates cardioprotection through p62 and mitophagy.

## Materials and Methods

All procedures involving mice were approved by the Animal Care and Use Committee at San Diego State University under Animal Protocol number 09-10-032G, and conform to the Guide for the Care and Use of Laboratory Animals.

### Cell Culture

HL-1 cardiomyocytes were maintained in Claycomb media (Sigma-SAFC Biosciences) as described previously [25]. MEF ATG5^+/+^ and ATG5^−/−^ cells obtained from RIKEN (Dr. Mizushima) [26] were maintained in DMEM (Invitrogen-Gibco).

### Cell Transfection and Gene Silencing

Transfections and gene silencing were performed using Effectene (Qiagen) according to the manufacturer's recommendation. Cells were transfected with mCherry-Parkin in Effectene for 4 hr, then used for experiments 18 hr later. p62 (sc-36108) and control scrambled (sc-37007) silencing complexes (100 nM) obtained from Santa Cruz Biotechnology were transiently transfected into HL-1 cardiomyocytes for 6 hr. After 36 hr, cells were harvested and post nuclear supernatants were analyzed by Western blot to confirm knockdown of p62. Parallel cell cultures were then subjected to simulated ischemia and compared to controls.

### *In Vitro* Simulated Ischemia

Cells were subjected to ischemia-mimetic solution containing [in mM: NaCl 125, KCl 8, 2-deoxyglucose 20, Na-Lactate 0.5, MgSO_4_ 1.25, CaCl_2_ 1.25, KH_2_PO_4_ 1.2, NaHCO_3_ 6.25, HEPES 20 (pH 7.4)]. Culture dishes were then placed into an anaerobic gas generating pouch system (Becton Dickson) which was then depleted of oxygen by forcing in a mixture of carbon dioxide and nitrogen (1∶19) before being sealed. Culture dishes undergoing simulated ischemia were incubated at 37°C for the indicated time before harvesting. Reperfusion media used was Krebs-Ringer Bicarbonate Buffer (Sigma K4002-1L) supplemented to [in mM:, NaHCO_3_ 25, CaCl_2_ 1, HEPES 10 (pH 7.4)].

### Cytotoxicity Assay

Media collected from cells after 60 min reperfusion were assayed for LDH release using the Tox7 *In Vitro* Toxicology Assay Kit (Sigma) according to the manufacturer's instructions. Results were normalized to the total LDH for each sample after cells were lysed.

### *In Vivo* Ischemic Preconditioning (IPC), Ischemia/Reperfusion and Infarct Size Measurement

Parkin (*Park2*) gene knockout mice (Parkin^−/−^) and control C57BL/6J mice were purchased from Jackson Laboratories. Coronary occlusion was performed as described previously [27]. Briefly, 10–12 week old *Parkin* knockout and control mice were anesthetized with an i.p. injection of ketamine (10 mg/kg) and xylazine (1 mg/kg). Isoflurane anesthesia was used throughout the procedure. Pressure-controlled ventilation (Harvard Apparatus) was maintained at a rate of 140 bpm and a pressure of 9 cm H_2_O. To induce IPC, a PE-10 tube was placed on the surface of the left anterior descending artery (LAD) which was ligated together with an 8-0 silk suture for 3 minutes followed by 3 minutes reperfusion for 3 cycles. For biochemical studies, hearts were excised immediately after IPC and put in cold PBS. Risk area of infarction was dissected out and frozen in liquid nitrogen. Sham-operated mice had a suture placed around the LAD that was not tightened for the length of the procedure. For infarct size determination, hearts were subjected to 20 minutes ischemia with or without IPC. The suture was released but left in position, and after verifying adequate reperfusion, the chest was closed and the mice were allowed to recover for 22 hr. Sham-operated mice were handled in parallel. After 22 hr, mice were anesthetized, the chest re-opened, and the hearts were injected with 1% Evans Blue and then harvested. Transverse 1 mm thick slices of the ventricles were stained in 1% triphenyltetrazolium chloride (Sigma).

### Langendorff Perfusion

The isolated perfused rat heart model was previously described [28]. In brief, after anesthesia and heparinization (pentobarbital sodium 60 mg/kg i.p. and heparin 500 U i.p.), rat hearts were excised into ice cold Krebs-Henseleit solution (in mM: NaCl 118.5 mM, KCl 4.7, KH_2_PO_4_ 1.18, MgSO_4_ 1.18, NaHCO_3_ 25, glucose 11.1, CaCl_2_ 2.5) and perfused with oxygenated buffer at constant pressure (60 mmHg) for 5 min and then subjected to IPC for 3 cycles of 5 minutes ischemia alternating with 5 minutes reperfusion. Hearts were further subjected to ischemia and reperfusion as indicated in figure legends. Tissues were harvested and immediately frozen in liquid nitrogen for later study.

### Isolation of Mitochondria

#### Cell Fractionation

Cells were harvested in (sucrose homogenization buffer containing (in mM): sucrose 250, EDTA 1, HEPES 10, pH 7.4 and freshly added Protease Inhibitor Cocktail Complete (Roche)). Cells were then disrupted by forcefully passing cells through a 27.5 gauge syringe four times in a microfuge tube. Mixtures were then spun at 600g for 5 minutes to eliminate the nuclear fraction and unbroken cells. Supernatants were then spun at 7000*g* for 10 minutes. Pellets were resuspended in homogenization buffer and centrifuged at 7000*g* for 10 min to obtain the final mitochondria-enriched pellet (heavy membrane fraction). Pellets were resuspended in homogenization buffer supplemented with 1% Triton X-100. *Tissue Fractionation:* Frozen heart samples were thawed on ice in MS buffer containing (in mM: mannitol 220, sucrose 70, EGTA 2, MOPS 5, pH 7.4, and freshly added Protease Inhibitor Cocktail Complete (Roche)). The tissue was then minced and homogenized by Polytron (Kinematica, Basel, Switzerland) on ice for 3 cycles of 15 s. The homogenates were centrifuged at 1000*g* for 10 min at 4°C, and the crude supernatants were further centrifuged at 3,000*g* for 15 min at 4°C. The mitochondria-enriched pellet was designated as heavy membrane fraction and the supernatant as crude cytosol. Samples were stored at −80°C until use.

### Immunofluorescence

#### Cells

Cells cultured in 35 mm glass bottom dishes (Mat-Tek) were fixed with 4% paraformaldehyde for 10 min then washed with 1× PBS 3 times 5 min. Cells were then blocked/permeabilized for 15 min in Tris-buffered saline (TBS)(in mM: NaCl 150, Tris-HCl 100, pH 7.4) supplemented with 5% horse serum, 5% goat serum, 0.2% BSA and 0.3% Triton X-100. After 3×5 min washes in TBS, cells were incubated 1 hr at 37°C with 1∶200 anti-Tom70 polyclonal rabbit antibody (Calbiochem), 1∶200 anti-Parkin mouse monoclonal antibody (SCBT), 1∶200 anti-p62 rabbit polyclonal antibody (MBL), or 1∶200 anti-COX4 mouse monoclonal (Invitrogen). After 3×5 min washes with TBS, cells were incubated with either anti-rabbit AlexaFluor-488-conjugated (Invitrogen), anti-rabbit AlexaFluor-594-conjugated (Invitrogen), or anti-mouse Texas Red-conjugated (Abcam) secondary antibodies for 30 min at room temperature in the dark. Cells were washed again as described above, and stored at 4°C in the dark immersed in 1× PBS. *Tissues:* Hearts were embedded in Optimal Cutting Temperature compound, and 7 µm frozen sections were prepared. For immunostaining, tissue sections were immersed in acetone for 10 min at room temperature and then allowed to air dry for 10 min. Samples were incubated in TBS supplemented with 5% horse serum, 5% goat serum, and 0.3% Triton X-100 for 20 min, and then incubated for 2 hr with primary antibody (1∶200 anti-Parkin from SCBT and 1∶500 anti-Tom70 from Calbiochem), followed by washing and staining with the appropriate fluorescent secondary antibodies. Cells and stained tissue sections were imaged on a Nikon TE300 fluorescence microscope equipped with a cooled charge-coupled device camera (Orca-ER, Hamamatsu). Deconvolution of images was performed using Autodeblur Software. Colocalization, correlation, and quantitation were performed using ImageJ software (NIH).

### Western Blot Analysis

Sample proteins from experiments were quantified using the Bio-Rad protein assay kit. Equal amounts of protein were then resolved on 10–20% Tris-Glycine SDS-PAGE gels (Invitrogen) and transferred to nitrocellulose membranes. The membranes were blocked with 5% nonfat dry milk for 1 hr then incubated with 1∶500–1∶1000 diluted primary antibodies against Tom70 (Calbiochem), p62 (BD), actin (Sigma), Parkin (SCBT), or COX3 (Molecular Probes) at 4°C overnight. Membranes were washed with TBS buffer with 1% Triton X-100 at room temperature and incubated with appropriate peroxidase-conjugated secondary antibodies (1∶2,500). Blots were developed with SuperSignal West Dura Extended Duration Substrate (Thermo-Pierce), and immunoreactive bands were visualized using the ChemiDoc XRS system (Bio-Rad). Densitometry was performed using ImageJ software.

### Statistical Analysis

Student's T-Test was used to determine statistical significance with p values less than 0.05 accepted as significant. Error bars indicate STDEV. ANOVA was used as indicated for multiple group analysis with error bars indicating SEM.

## Results

### Parkin translocates to mitochondria in response to simulated ischemia

**Figure 1:**
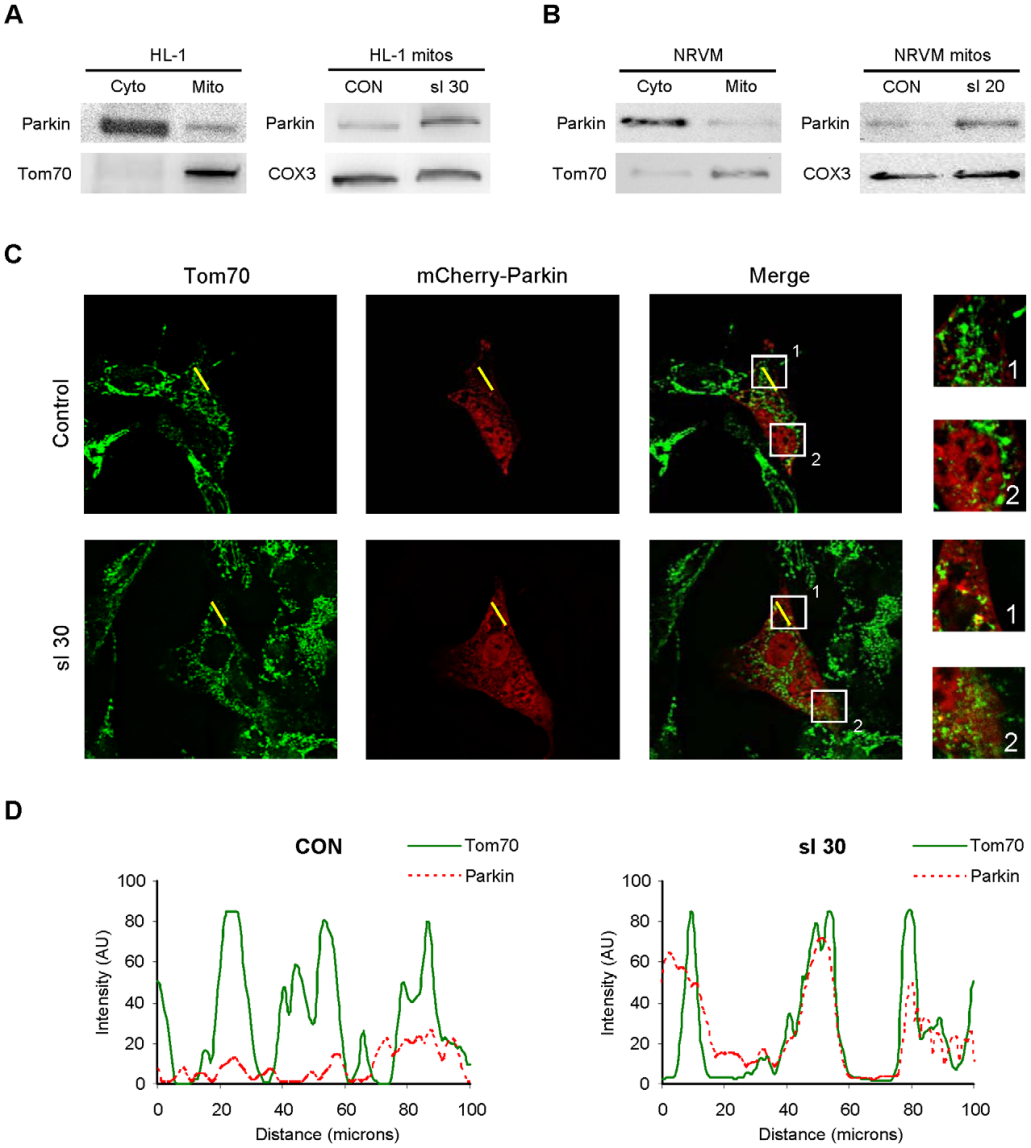
Parkin redistributes to mitochondria in cardiomyocytes subjected to simulated ischemia (sI). **A.** HL-1 cells were subjected to simulated ischemia (ischemia-mimetic buffer and hypoxia) for the indicated time, then fractionated to yield cytosol and heavy membranes (mitochondria-enriched fraction). Right-hand panel shows Parkin in the heavy membrane fraction under basal conditions (CON) and after 30 min of simulated ischemia (sI 30). **B.** Neonatal rat ventricular cardiomyocytes were subjected to simulated ischemia, then fractionated to yield crude cytosol and heavy membrane fractions. Right-hand panel shows Parkin in the heavy membrane fraction under resting conditions (CON) and after 20 min sI. **C.** HL-1 cells transfected with mCherry-Parkin (red) were subjected to 30 min sI, then fixed and immunolabeled with mitochondrial marker Tom70 (green). Yellow line indicates the segment used for pseudo-line scan analysis. Boxes indicate regions enlarged at right. Images are representative of 5 independent replicates. **D.** Pseudo-line-scan tracing indicates distribution of mitochondria (Tom70, solid green line) and mCherry-Parkin (dotted red line).

Our previous work demonstrated a role for autophagy in the cardioprotection afforded by ischemic preconditioning (IPC) [29]. Mitochondria are one target of autophagy, and a number of groups have shown that Parkin is important for mitophagy in neuronal tissues. The role of Parkin in the heart has not been assessed, although cardiac expression of Parkin has been described [11]. Under resting conditions, Parkin was present in the cytosol in HL-1 cells and neonatal rat cardiomyocytes. However, in response to the stress of simulated ischemia (sI), endogenous Parkin translocated to mitochondria (**Fig. 1A and B**).

To further investigate the redistribution of Parkin, we transected HL-1 cells with mCherry-Parkin and assessed its colocalization with mitochondria before and after sI (**Fig. 1C**). Pseudo-line scan analysis confirmed that mCherry-Parkin translocated to mitochondria after sI (**Fig. 1D**). Mitochondrial fragmentation was also apparent after sI; notably, Parkin was associated with only a subset of mitochondria (**Fig. 1C** **, insets**).

### Parkin translocates to mitochondria in response to ischemic preconditioning

**Figure 2:**
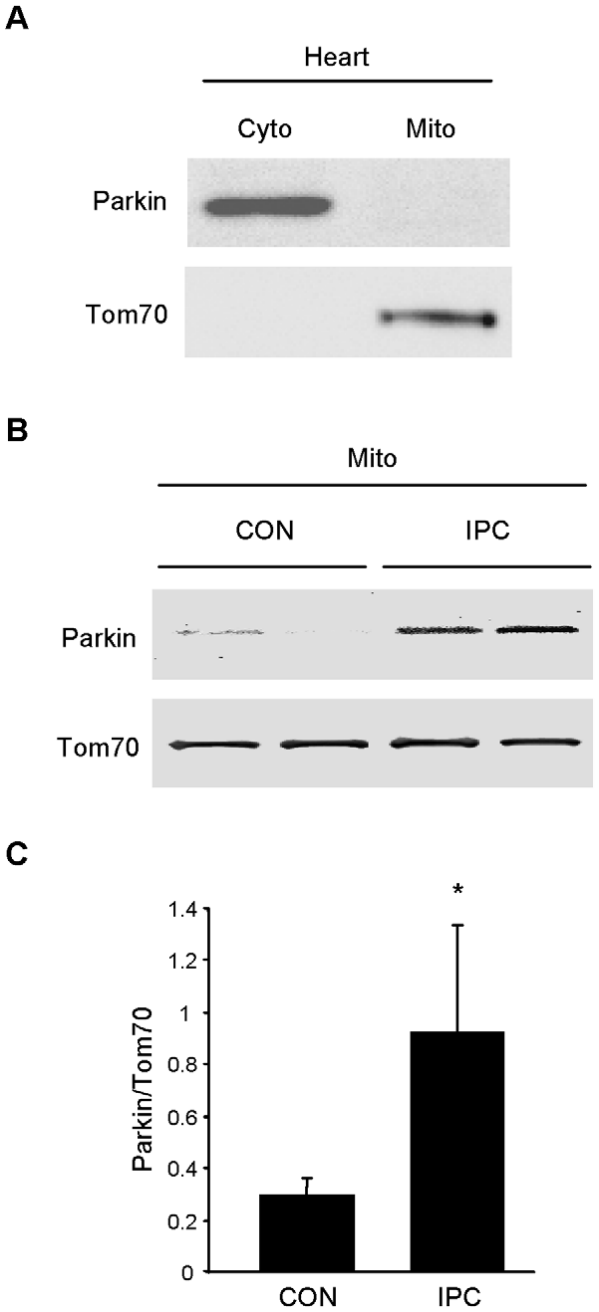
Parkin translocates in response to IPC in rat hearts. **A.** Under resting conditions, Parkin is restricted to cytosol in the heart. **B.** Langendorff-perfused rat hearts were subjected to continuous perfusion (CON) or 3 cycles of 5 min global no-flow ischemia alternating with 5 min reperfusion (IPC). Immediately after the third 5 min reperfusion, hearts were homogenized and fractionated to yield crude cytosol and heavy membrane fractions which were probed for Parkin. **C.** Quantification of Parkin in mitochondrial fractions from CON and IPC hearts is shown (*p<0.05, n = 4).

**Figure 3:**
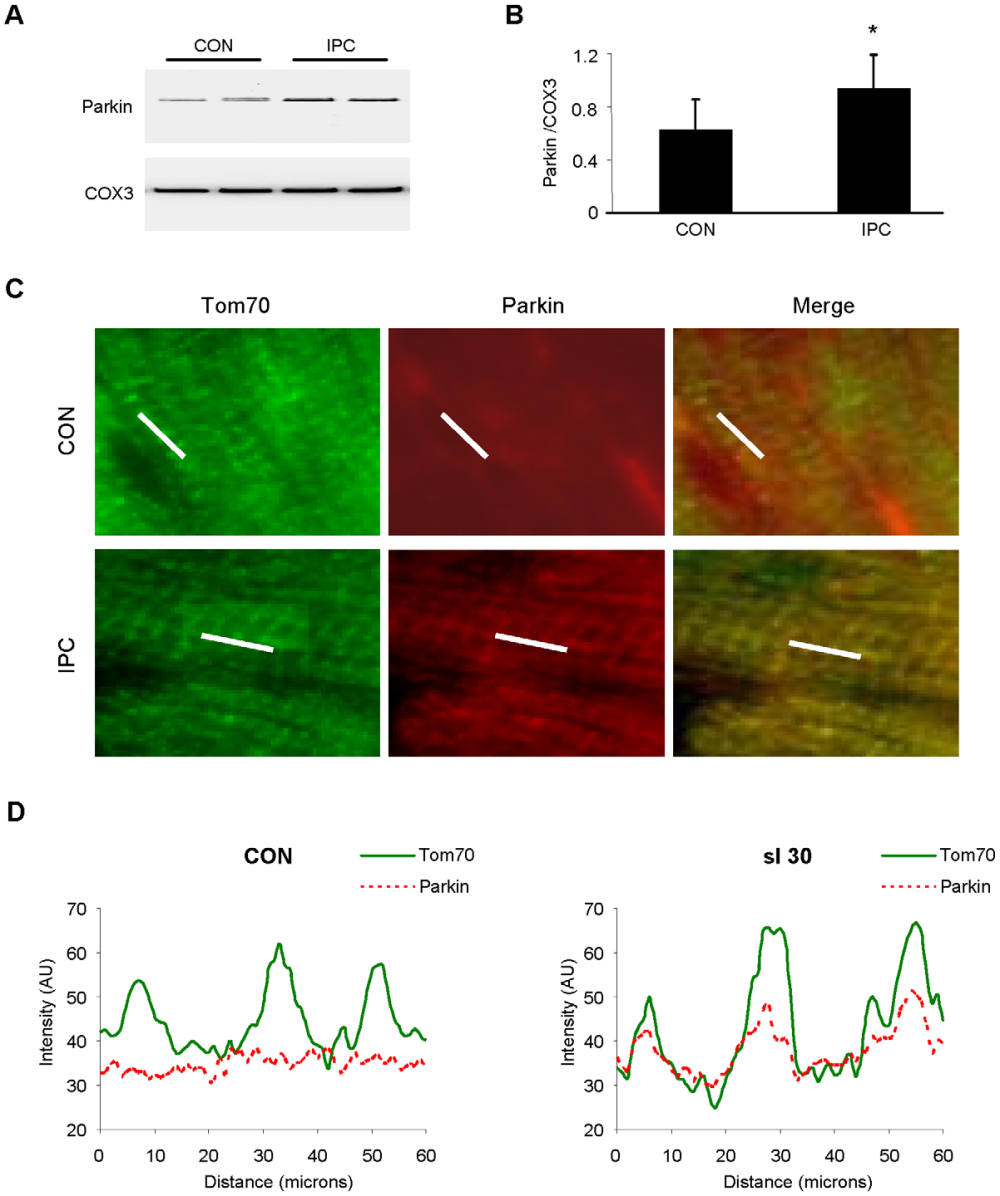
IPC triggers Parkin translocation to mitochondria in vivo. **A.** Mice were subjected to IPC consisting of 3 cycles of 5 min coronary artery ligation alternating with 5 min reperfusion. Heavy membrane fractions prepared from heart were probed for Parkin. Cytochrome oxidase subunit III (COX3) was used to normalize mitochondrial loading. **B.** Quantification of Parkin translocation to mitochondria is shown (*p<0.05, n = 5). **C.** Mice were subjected to 3 cycles of IPC in vivo. Cryosections of hearts were examined to visualize Parkin translocation to mitochondria by immunolabeling with antibodies to Tom70 (green) and Parkin (red). White line indicates segment used for pseudo-line scan analysis. Representative images from the risk zone of sham-operated (CON) and preconditioned (IPC) hearts are shown. **D.** Pseudo-line scan analysis of mitochondria (Tom70, solid green line) and endogenous Parkin (dotted red line) in cryosections from control (CON) and preconditioned (IPC) mouse hearts demonstrates translocation of Parkin to the mitochondria after IPC.

We next sought to determine if Parkin translocated to mitochondria in response to IPC. Under resting conditions, Parkin was detected only in the crude cytosol fraction (**Fig. 2A**). Langendorff-perfused rat hearts subjected to continuous perfusion or 3 cycles of 5 min global no-flow ischemia alternating with 5 min reperfusion were homogenized to yield heavy membrane and crude cytosol fractions. Western blotting revealed Parkin translocation to the heavy membrane fraction (**Fig. 2B and C**). These findings were further confirmed in vivo in mice subjected to IPC by western blot analysis of cytosol and mitochondria (**Fig. 3A and B**). We also verified Parkin translocation by immunofluorescence microscopy of cryosections of heart tissue (**Fig. 3C**). While distribution of Parkin was non-specific in sham-operated hearts, IPC induced redistribution of Parkin to mitochondria, as assessed by pseudo-line scan colocalization analysis of deconvolved images (**Fig. 3D**). Taken together, these results indicate that Parkin translocates to mitochondria in response to ischemic preconditioning.

### Parkin translocates to depolarized mitochondria

**Figure 4:**
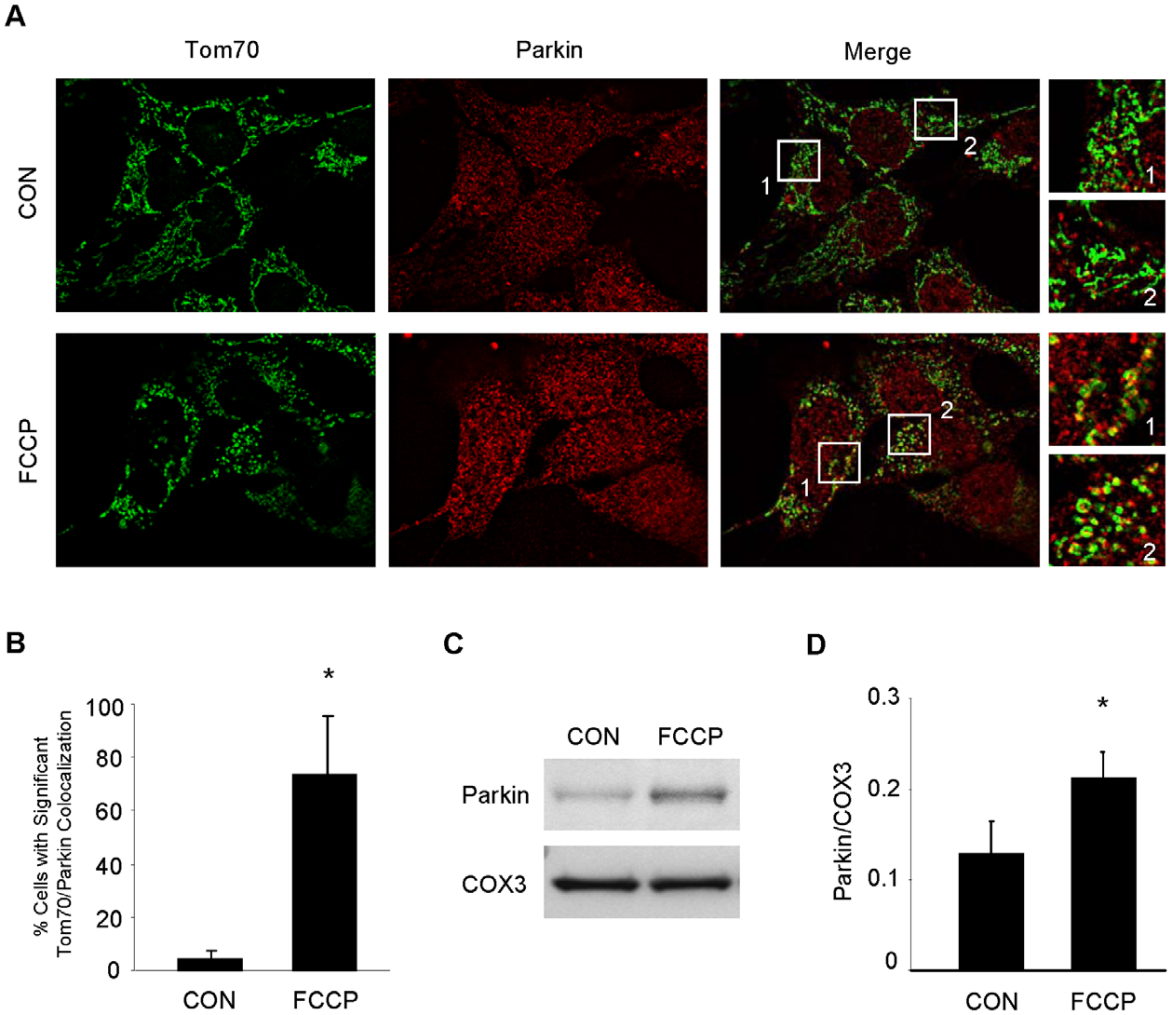
FCCP induces Parkin translocation to mitochondria in HL-1 cardiomyocytes and mitochondrial fractions in Langendorff-perfused rat hearts. **A.** HL-1 cardiomyocytes were treated with the mitochondrial uncoupler FCCP (10 µM) or vehicle (ethanol, CON) for 1 hour, then fixed and immunolabeled for Tom70 (green) and Parkin (red). Boxes outline fields that were enlarged (at right) to show details of mitochondrial structure and colocalization. **B.** Cells were then scored for significant colocalization. Over 100 cells from sequential fields were assessed for each group (*p<0.03, n = 4). **C.** Isolated rat hearts were subjected to continuous perfusion with 100 nM FCCP or vehicle (ethanol, CON) for 5 minutes after stabilization with KHB. Mitochondrial fractions were probed for Parkin. **D.** Quantification of Parkin in mitochondrial fractions from CON and FCCP treated hearts (*p<0.05, n = 4).

Parkin translocates to depolarized mitochondria [6], [30]. Mitochondrial depolarization has been implicated as a mechanism of cardioprotection mediated by diazoxide [31] and low-dose FCCP [32]. We confirmed that FCCP induced Parkin translocation in HL-1 cells exposed to 10 µM FCCP or vehicle for 1 hr (**Fig. 4A and B**). To determine if low-dose FCCP mediated Parkin translocation in the heart, we perfused rat hearts in Langendorff mode with 10 nM FCCP or vehicle for 5 min, and assessed Parkin distribution in cytosol and mitochondrial fractions (**Fig. 4C and D**). In cells and perfused hearts, mitochondrial depolarization after FCCP triggered translocation of Parkin to mitochondria.

### Ischemia/IPC-induced p62 translocation to the mitochondria is dependent on Parkin

**Figure 5:**
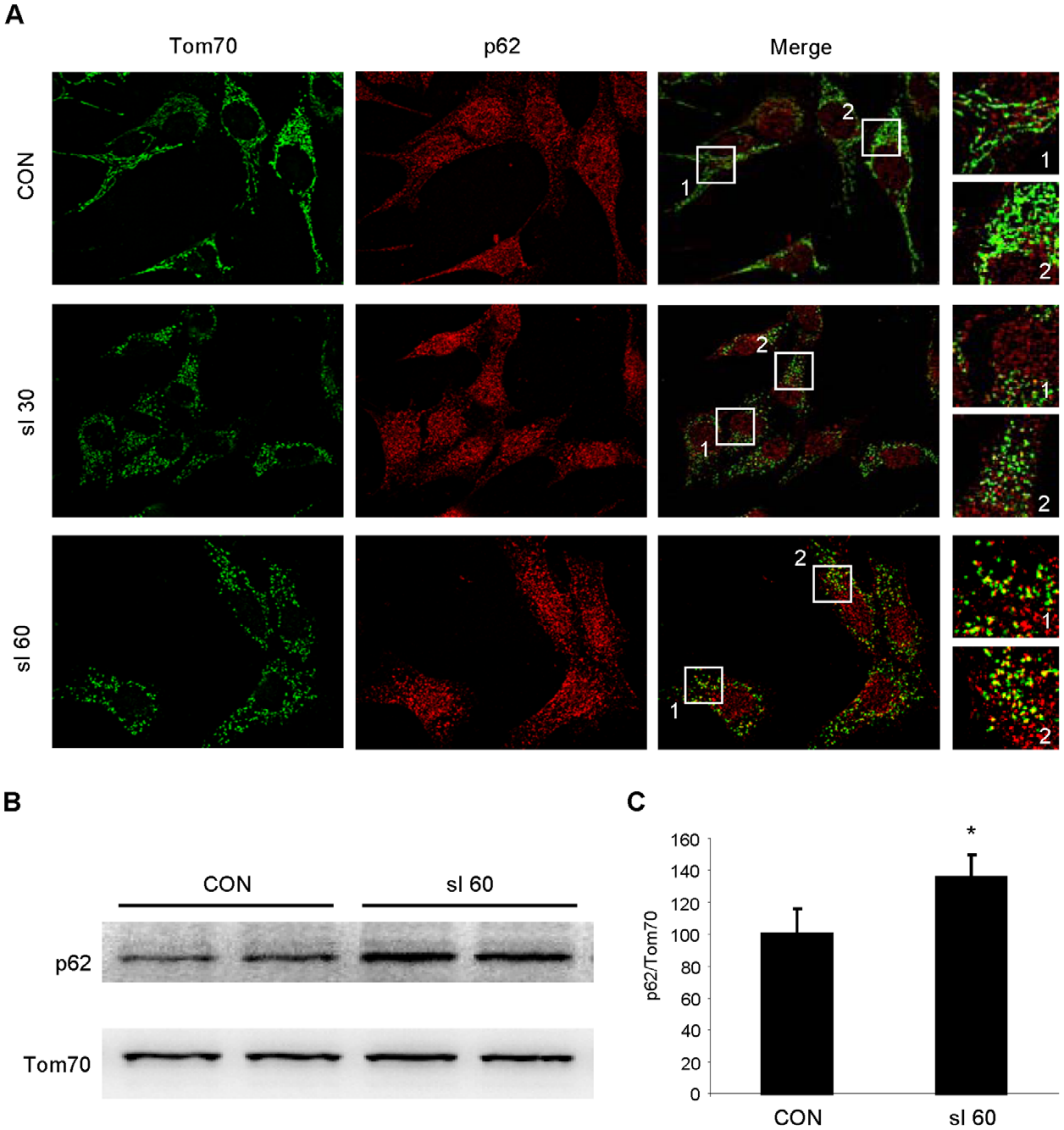
p62 translocates to mitochondria in HL-1 cells subjected to simulated ischemia (sI). **A.** HL-1 cells were subjected to sI for 30 or 60 min, then fixed and immunolabeled for Tom70 (green) and p62 (red). Boxes outline fields that were enlarged (at right) to show details of mitochondrial structure and colocalization. **B.** p62 Western blot of mitochondrial fractions from HL-1 cardiomyocytes subjected to 60 min sI. **C.** Quantification of Western blots is shown (Student's T-test: *p<0.02, n = 3).

**Figure 6:**
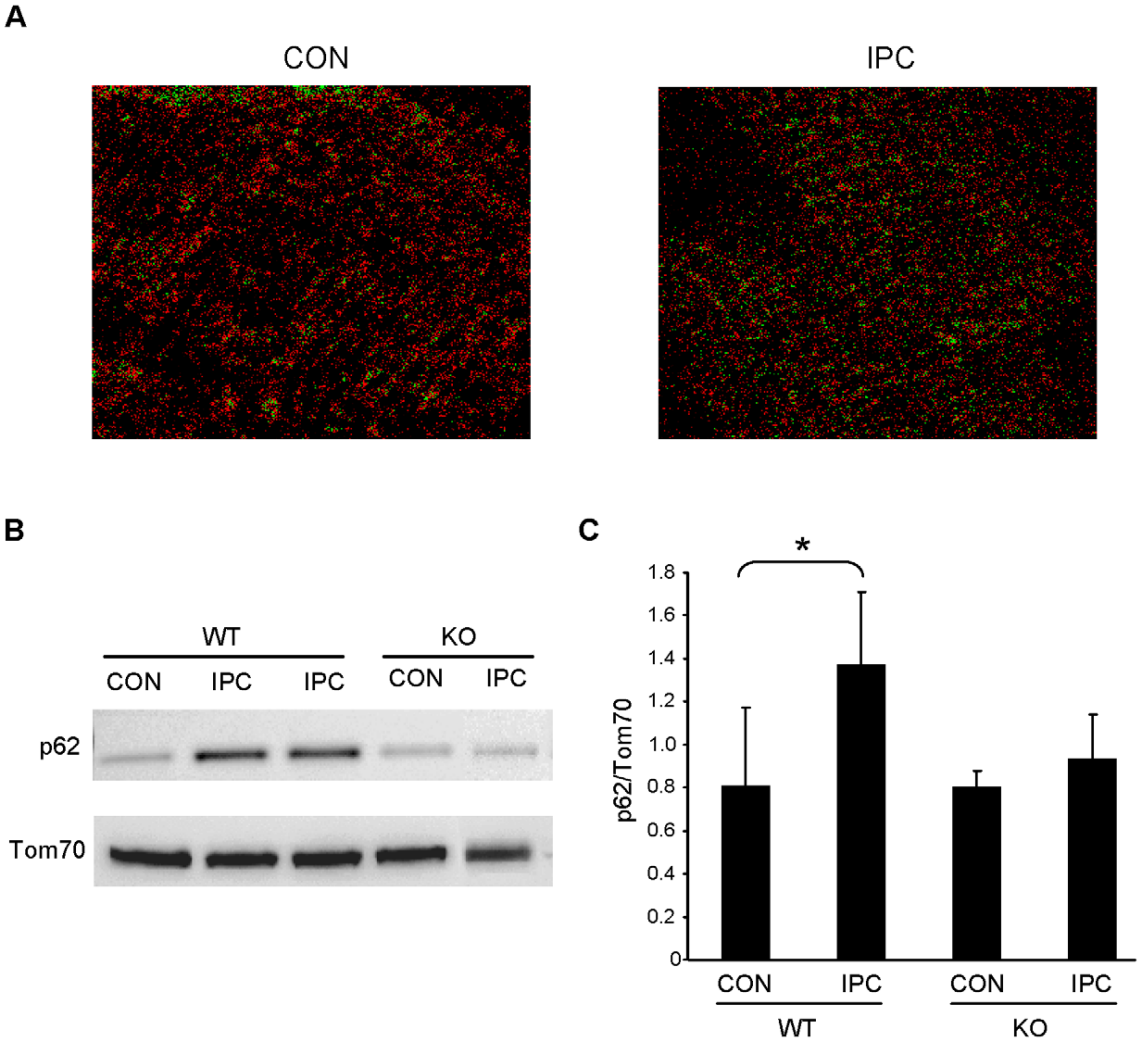
Translocation of p62 to mitochondria is induced by IPC. **A.** Cryosectioned hearts from WT mice sham operated (CON) or subject to IPC were immunolabeled for p62 and Tom70. Colocalization was assessed via PDM value images using Image J software where high colocalization scores are shown in green. **B.** WT and Parkin^−/−^ (KO) mice were subjected to sham surgery (CON) or IPC, and mitochondria were probed for p62 and normalized to Tom70. **C.** Quantification of p62 translocation is shown (*p<0.05, n = 5).

Previous studies have demonstrated that p62 recognizes and binds ubiquitinated proteins and recruits autophagosomes via its LC3-binding domain [33]. In HL-1 cells and neonatal cardiomyocytes, we observed that p62 translocated to the mitochondria in response to simulated ischemia of 30 and 60 min. This was demonstrated by immunofluorescence (**Fig. 5A**) and by Western blotting of subcellular fractions (**Fig. 5B and C**). To determine if Parkin is important in vivo for IPC-induced p62 recruitment to the mitochondria, we assessed p62 translocation after IPC in wild type and Parkin knockout mice. Following IPC, p62 translocation to the mitochondria increased in the hearts of wild type mice, but not in Parkin knockout mice, based on immunolabeling of cryosections with colocalization analysis (**Fig. 6A**) and immunoblot analysis of heavy membrane fractions (**Fig. 6B and C**). These data strongly suggest that Parkin is essential for mitochondrial translocation of p62 in response to ischemic stress or IPC.

### Parkin and p62 mediate mitophagy in response to ischemic stress

**Figure 7:**
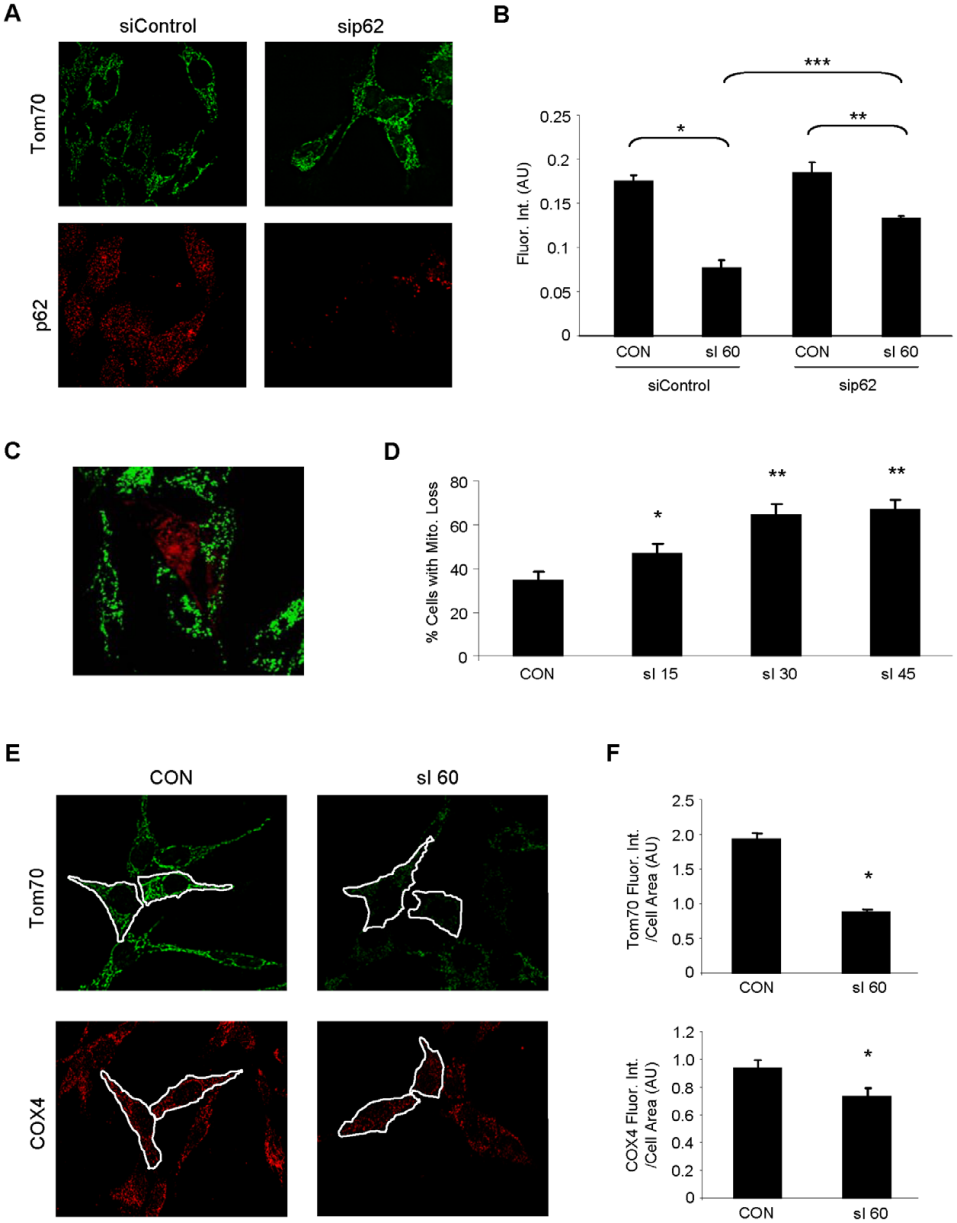
Depletion of p62 attenuates sI-induced mitochondrial loss. **A.** HL-1 cells were treated with scrambled siRNA (siControl) or siRNA corresponding to p62 (sip62) and labeled with antibodies to Tom70 (green) and p62 (red). **B.** HL-1 cells treated with siRNA were subjected to sI for 60 min and mitochondrial mass was quantified by fluorescence intensity of the mitochondrial marker Tom70. Over 100 cells were assessed for each group and the experiment was performed 3 times. Error bars represent SEM of the 3 experiments (ANOVA: *p<0.001, **p<0.05, ***p<0.005). **C.** Shown is a representative deconvolved image of HL-1 cells transfected with mCherry-Parkin (red) and subjected to sI for 30 min and probed for Tom70 (green). **D.** Mitochondrial content in mCherry-Parkin-transfected cells was assessed by fluorescence intensity of Tom70, and the percentage of cells showing substantial mitochondrial loss (similar to the cell depicted here in the center of the field) was scored for non-ischemic cells (CON) and time points of 15, 30 and 45 min of simulated ischemia. A minimum of 100 transfected cells were scored for each time point (ANOVA: *p<0.01, **p<0.001 versus CON, n = 4). Error bars represent SEM of the 4 experiments. **E.** Mitochondrial content per cell was assessed in HL-1 cardiomyocytes subjected to 60 min of sI. The total amount of green (Tom70, outer mitochondrial membrane marker), and red (COX4, inner mitochondrial membrane marker) fluorescence intensity per unit area within each cell were measured (ImageJ). **F.** For each condition, 100 cells were scored in sequential fields (*p<0.01, n = 4).

Mitochondria are a frequent target of autophagy. To determine the effects of simulated ischemia on mitochondrial loss, mitochondrial content was compared in HL-1 cells treated with siRNA to downregulate p62/SQSTM1 or a control siRNA. Simulated ischemia resulted in substantial mitochondrial loss which was attenuated when p62 was downregulated (**Fig. 7A and B**). HL-1 cells were transfected with mCherry-Parkin and subjected to simulated ischemia for 15–45 min. The percentage of cells showing mitochondrial depletion increased with the duration of simulated ischemia and was much more pronounced in the mCherry-Parkin-expressing cells (**Fig. 7C–F**). However, mitophagy was not simply an artifact of overexpression of mCherry-Parkin, as mitochondrial content per cell also diminished in non-transfected cells subjected to simulated ischemia. To determine if the disappearance of mitochondria was a consequence of mitochondrial outer membrane degradation, we repeated the experiments using an antibody to COX4 (**Fig. 7E and F**). We saw a consistent reduction in both mitochondrial antigens in Parkin-transfected cells.

**Figure 8:**
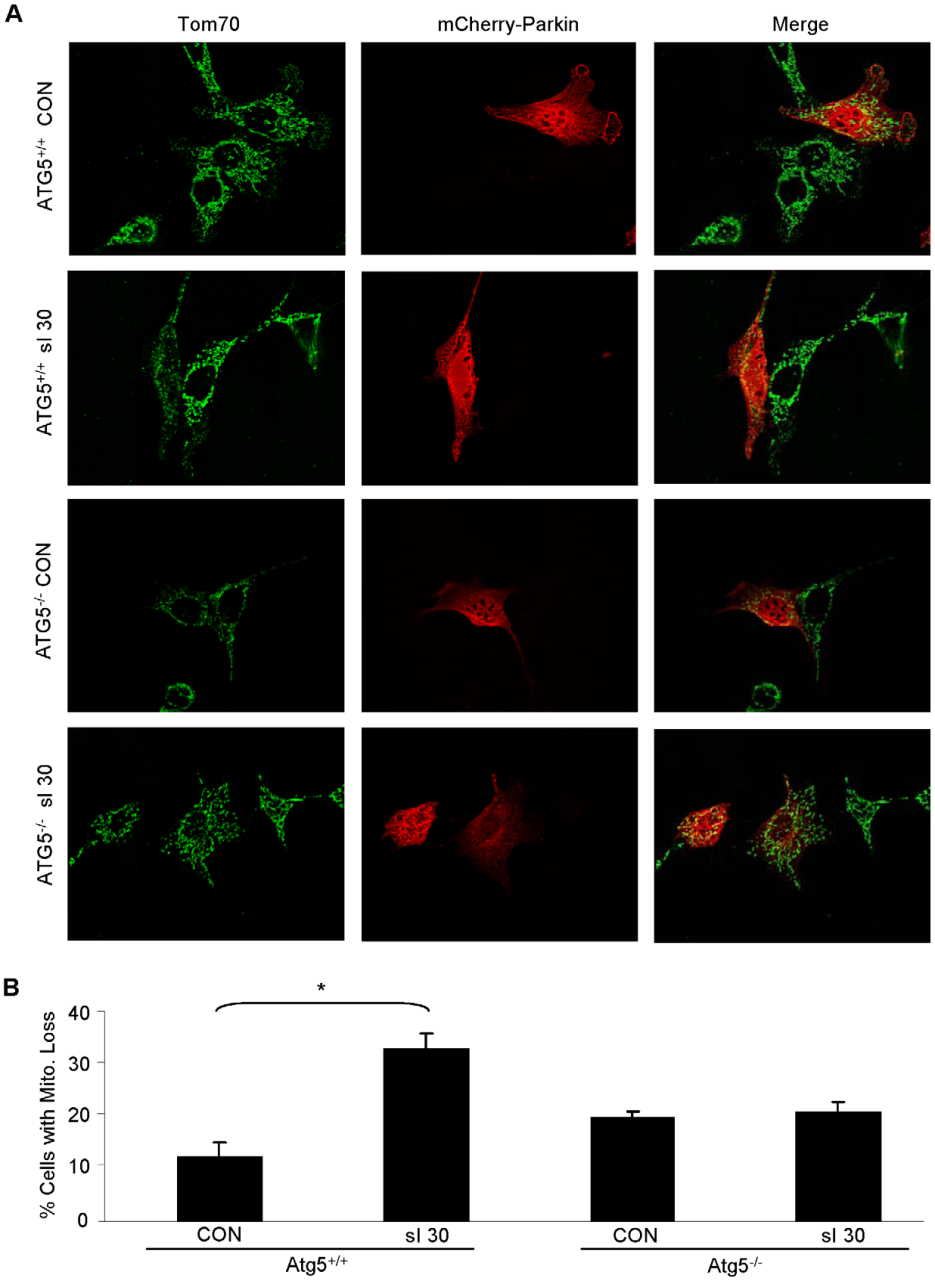
Autophagy is necessary for selective mitochondrial loss mediated by Parkin after simulated ischemia. **A.** MEFs from ATG5^+/+^ and ATG5^−/−^ mice were transfected with mCherry-Parkin (red) and then subjected to 30 min sI. After fixation, mitochondria were visualized with antibodies to Tom70 (green). Representative deconvolved images are shown. **B.** Mitochondrial depletion was quantified in a minimum of 50 mCherry-Parkin expressing cells per condition (*p<0.01, n = 4).

In order to determine if the mitochondrial loss observed following simulated ischemia is an autophagy-mediated process, we employed the use of Atg5^−/−^ MEFs. Previous studies have shown that the ability of Atg5^−/−^ MEFs to induce autophagy is severely blunted [34]. Indeed, following 30 min of simulated ischemia, wild-type MEFs exhibited a marked increase in the percentage of cells that had lost significant mitochondrial mass; similarly, the average mitochondrial content per cell was unchanged after simulated ischemia in the Atg5^−/−^ MEFs (**Fig. 8A and B**). Combined, these results strongly suggest that Parkin and p62 play an essential role ischemia-induced mitophagy.

### Parkin protects myocytes against ischemia/reperfusion injury

**Figure 9:**
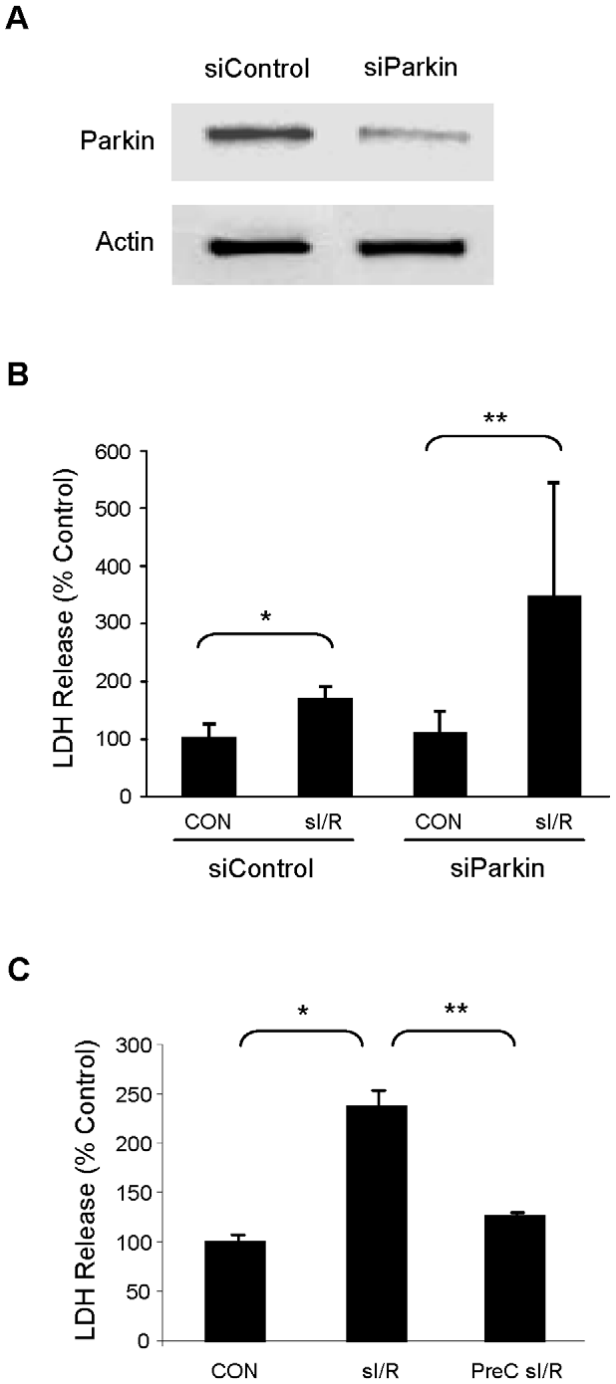
Parkin mediates cytoprotection in HL-1 cells subjected to simulated ischemia and reperfusion (sI/R). **A.** HL-1 cells were treated with siRNA for Parkin or control siRNA and lysates were probed for Parkin protein. Equal protein loading is shown by blotting for actin. **B.** Cells treated with siRNA were subjected to sI/R (60 min simulated ischemia and 60 min reperfusion) and cell death was determined by measuring LDH release into the culture medium (Student's T-Test: *p<0.05, **p<0.001, n = 6). **C.** HL-1 cells were incubated in normal media for 60 min, subjected to sI/R, or preconditioned (PreC) with 30 min sI and 60 min recovery in normal media before being subjected to sI/R. Cell culture supernatants during the final 60 min reperfusion were assayed for LDH release to determine cell death. (*p<0.005, and **p<0.01, n = 3).

Having established the requirement for autophagy in IPC, and having demonstrated the pivotal role of Parkin in mediating mitophagy in response to ischemic stress, we sought to determine if Parkin plays a protective role. We used siRNA to downregulate Parkin in HL-1 cardiomyocytes (**Fig. 9A**) and subjected them to 60 min simulated ischemia and 60 min reperfusion (sI/R). Parkin knockdown significantly increased the extent of cell death after sI/R, as assessed by LDH release (**Fig. 9B**). In contrast, 30 min simulated ischemia was not associated with LDH release (data not shown), but was sufficient to induce a protected state when followed by the 60 min index ischemia and reperfusion, as indicated by attenuated LDH release (**Fig. 9C**).

**Figure 10:**
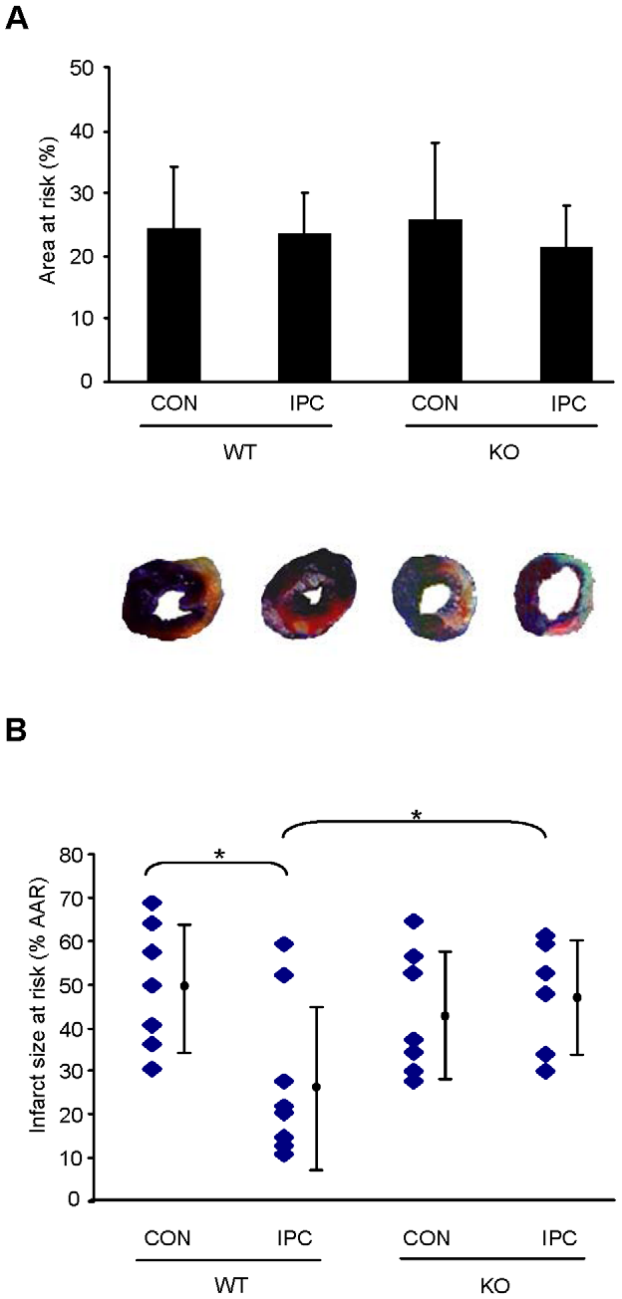
Parkin is required for IPC. **A.** WT and Parkin^−/−^ (KO) mice were subjected to sham treatment (CON) or IPC, followed by 20 min ischemia and 22 hr reperfusion. Area at risk was determined by Evan's blue injection after re-occlusion and showed no difference between groups. **B.** Infarct size was determined by TTC staining and reported as % of the area at risk. IPC reduced infarct size in WT mice but not in Parkin KO mice (*p<0.05, n = 6). Representative heart slices are shown.

To determine whether Parkin is required for cardioprotection by IPC *in vivo*, we subjected Parkin knockout mice to IPC, followed by ischemia/reperfusion and determination of infarct size. Ablation of Parkin abolished IPC-induced cardioprotection (**Fig. 10**), thus revealing the important role of Parkin in cardioprotection by IPC.

## Discussion

Parkin has been shown to play a central role in mitochondrial quality control in the brain, but its role in heart has not been examined to date, although both organs consist of long-lived cells with a high mitochondrial content. Because we have previously shown a role for autophagy in cardioprotection by IPC, we considered whether mitochondria were an important target of autophagy; in particular, we considered whether Parkin might play an important role in mitophagy in the setting of IPC. The results presented herein reveal an important role for Parkin and p62 in mediating mitophagy in response to ischemic stress in HL-1 cells, and in mediating IPC-dependent cardioprotection in vivo.

Studies dating back to the 1970s have described the occurrence of mitochondria in autophagosomes in heart tissue [35], [36]. More recently, the mitochondrial permeability transition pore has been implicated in signaling autophagy [37], but the mechanism by which cardiac mitochondria might be targeted for selective removal has not been elucidated. We have shown that HL-1 cells undergo cyclophilin D-dependent mitophagy in response to starvation [24], but this is the first study to examine the processes mediating mitophagy in response to ischemic stress in the heart.

Why would removal of mitochondria be beneficial? Selective removal of the mitochondria with the lowest threshold for opening of the mitochondrial permeability transition pore (MPTP) would leave behind a population of robust mitochondria better-equipped to resist ischemic stress. Since depolarized mitochondria will hydrolyze ATP in a futile effort to restore membrane potential, elimination of these mitochondria would actually result in increased ATP content after I/R despite the reduced numbers of mitochondria. Selective removal of damaged mitochondria might also reduce the production of reactive oxygen species (ROS). Although we did not measure ROS production in this study, we previously reported that sulfaphenazole attenuates ROS production and protects the heart via autophagy, and that stimulation of autophagy attenuates ROS production by HL-1 cells exposed to lipopolysaccharide [38], [39].

The limitations of this study are that we have not measured PINK1, the mitochondrial outer membrane kinase that is stabilized as membrane potential decreases, nor have we measured mitochondrial membrane potential concurrently with Parkin or p62 translocation. We saw a consistent reduction in both mitochondrial antigens in Parkin-transfected cells. Although the magnitude of reduction was greater for Tom70 than COX4, the immunostaining for COX4 was done on a different set of cells, non-concurrently. Further work will be needed to determine if outer membrane and inner membrane antigens disappear concurrently—consistent with organellar destruction—or if outer membrane proteins are selectively degraded as suggested by Chan et al. [40]. We expect that mitophagy is selective and self-limited since the process is restricted to depolarized mitochondria [41], [42]. Global depolarization of mitochondria with an uncoupler such as FCCP is known to trigger generalized mitophagy and extensive loss of mitochondria [43]. Cells that eliminate mitochondria will need to replace them in order to fully restore ATP production capacity. Thus it will be important to assess mitochondrial biogenesis in future studies. Since biogenesis likely counterbalances mitophagy, it will be important to develop better methods to measure mitochondrial turnover in vivo.

The evidence presented in this study indicates that Parkin triggers mitophagy and mediates cytoprotection in HL-1 cells subjected to ischemic stress. These findings, coupled with the fact that IPC is abolished in Parkin knockout mice, lead us to conclude that Parkin mediates IPC through ubiquitination of mitochondrial membrane proteins and recruitment of p62 to facilitate autophagic engulfment of the decorated mitochondria.

The autophagic removal of depolarized mitochondria mediated by Parkin and p62 may represent an important element of mitochondrial quality control. Loss-of-function mutations of Parkin are linked to some cases of Parkinson Disease and are associated with mitochondrial dysfunction [44], [45]. While the role of Parkin in the heart is as yet unknown, it is interesting to note that the incidence of heart failure and coronary artery disease is doubled in elderly patients with Parkinson Disease compared to age-matched peers [46].
